# Equilibrium Frequency of Endosymbionts in Multiple Infections Based on the Balance between Vertical Transmission and Cytoplasmic Incompatibility

**DOI:** 10.1371/journal.pone.0094900

**Published:** 2014-04-18

**Authors:** Yuuki Kawasaki, Hiroshi Ito, Hisashi Kajimura

**Affiliations:** 1 Graduate School of Bioagricultural Sciences, Nagoya University, Nagoya, Japan; 2 Faculty of Design, Kyushu University, Fukuoka, Japan; University of Innsbruck, Austria

## Abstract

Cytoplasmic incompatibility (CI)-inducing endosymbiotic bacteria, such as *Wolbachia* and *Cardinium*, have been well studied through field data and validations on the basis of numerical simulations. However, the analytically derived equilibrium frequency of multiple infections has not yet been determined, although the equilibrium for cases of single infection has been reported. In this study, we considered the difference equation for endosymbionts using three parameters: the probability of the failure of vertical transmission (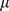), CI strength (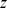), and the level of host inbreeding (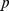). To analyze this model, we particularly focused on 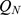, i.e., the frequency of host individuals completely infected with all 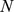-bacterial strains in the population. 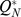, 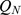 at the equilibrium state, was analytically calculated in the cases where 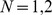 and 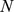 is any arbitrary value. We found that 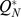 can be described using two parameters: 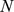 and 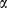, which is identical to 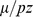. 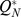 has a larger value in a system with a smaller 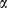. In addition, 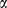 determines the maximum number of strains that infect a single host. Our results revealed the following: i) three parameters can be reduced to a single parameter, i.e., 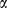 and ii) the threshold of the maximum number of infections is defined by 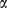, which prevents additional invasions by endosymbionts.

## Introduction

Endosymbiotic bacteria such as *Wolbachia* and *Cardinium* are well known to be reproductive manipulators infceting insect cells [1], [2]. Cytoplasmic incompatibility (CI) is considered to be the most common and efficient form of manipulation that can spread an infection throughout the host population. CI causes males infected with a bacterial strain reproductively incompatible with uninfected females [3]. Because of CI, infected females exhibit a relatively higher fitness than uninfected females; therefore, the number of infected individuals gradually increases and they become dominant in the host population. For example, approximately only 10% of *Drosophila simulans* individuals were infected with *Wolbachia* in the middle of the 1980s in California; however, the infection rate increased to 95% in 1993 [4], [5]. In a rearing system of *Encarsia pergandiella* infected with *Cardinium*, the infection gradually spread to almost fixation within the population, irrespective of the initial rate of infection [6].

The dynamics of CI-inducing bacteria can be primarily determined by three parameters: vertical transmission efficiency, fecundity of infected females, and CI levels [7], although the dynamics may be affected by other parameters [8], [9]. Vertical transmission, from a mother to her offspring, is the main route of transmission of the bacteria to other host individuals. The transmission rate is defined as the proportion of offspring infected from an infected mother, which is approximately 1 (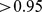 in most cases) [10]. Successful transmission has crucial effects on the spread and maintenance of the bacteria [11]. CI-inducing bacteria sometimes affect the fitness of the infected host diretctly through fecundity. The direction of their effect through fecundity depends on the host-bacteria combination, and the effect may be negative [12] or positive [13], or often neutral [14]. The CI level is defined as the proportion of the number of offspring died because of CI relative to the number of total offspring reproduced. In addition, the effect of CI is dependent on the host-bacteria combination, which highly varies from zero to one, i.e., from nearly neutral to complete mortality [15]–[17].

Models have been developed to study the dynamics of CI-inducing bacterial symbionts, particularly in *Wolbachia*, using the three conventional parameters [4], [18]. The long-term behavior depends on the initial frequency of bacterial infection [19]. If the initial frequency of bacterial infection is below the threshold, the frequency heads towards extinction; however, if the frequency stochastically exceeds the threshold (e.g., because of random genetic drift), then it is expected to spread to another equilibrium state where both infected and uninfected individuals exist. A higher vertical transmission rate and/or stronger CI will lead to higher infection frequency equilibrium. In most of the models on the basis of difference equations, the equilibria have rarely been analytically derived [20]–[22]. A few analytical results have been reported for single [7] and double infections [23]. Farkas & Hinow [23] analytically examined the case of hosts infected with only a single bacterial strain when two strains were present in the field. However, the symbiotic bacterial dynamics of an arbitrary number of strains remains poorly understood [7], [18], which prompts the questions: “how do the parameters determine the dynamics of infections and how many strains can infect a host population”? Experimental studies have reported multiple infections of *Wolbachia* in parasitic ants (nine strains) [24], byturid beetles (five strains) [25], and ambrosia beetles (five) [26]. However, whether a host population can be infected with a larger number of species is not known. Thus, the generalization of bacterial numbers is a big issue in endosymbiont studies.

In addition, the modeling of endosymbiotic dynamics in the context of mating system has not been extensively studied. CI is a phenomenon that occurs via mating, thus it is considered that the mating system should affect the dynamics of the endosymbionts. Mating systems can be categorized roughly as random mating and inbreeding. Random mating or panmictic mating is mating between any individuals in the population. In contrast, inbreeding or sib mating, is mating between a brother and a sister. Therefore, CI should be less effective under inbreeding because the mating partner should have the same infection status. Strong inbreeding has been observed in some insects, such as fig wasps and ambrosia beetles, which are known to have *Wolbachia* infections [26], [27]. Some theoretical studies have addressed the dynamics under inbreeding [28]–[30]. However, most of these models were examined numerically rather than analytically. In addition, the scope of these models was limited in the case of single or double infections.

This study aimed to develop an analytically solvable model of the infection dynamics of CI-inducing bacteria in sib/panmictic mating systems. We derived the analytical equilibrium of single and double infections, and generalized arbitrary numbers of strain infections using three parameters (vertical transmission rate, CI levels and the level of inbreeding). Our results show that bacterial infection thresholds can be simply expressed as a function of the ratio between the failure of vertical transmission and the CI level. In addition, we derived the maximum number of bacterial strains that are capable of infecting a host population.

## Methods

### Model

We consider a host population infected with 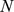 endosymbiotic strains. The integer 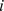 (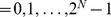) represents the state of each host individual, infected or uninfected with bacteria, where 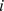 is written in base 2 with 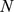 bits and each bit represents the state of infection with bacteria, i.e., 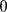 and 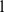 indicate absence and presence, respectively. For example, when 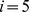 ( = 101 in base 2), the host is infected with the first and third bacterial strains but uninfected with the second strain. For convenience, we introduce a binary function 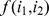, which indicates whether the set of infected bacterial strains 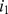 is included in that of 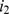. If the set of 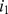 is a subset of that of 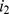, 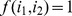, otherwise 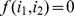. For example, 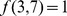 because the set of strains 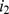 ( = 111 in base 2) contains any strains of 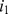 ( = 011 in base 2). In addition, we define 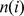 as the number of strains carried by an individual of type 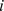.

We develop an difference equation to express the dynamics of the frequencies of hosts infected with multiple bacterial strains. Let 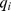 be the frequency of individuals infected with the bacterial set 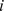. For simplicity, we assume that all of the bacteria have identical parameters in terms of the vertical transmission rate and CI strength among strains.

Next, we consider the formulation of CI among strains. CI results in a decreased hatching rate by a factor 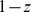; therefore, the fitness of individual mating incompatibly is reduced to 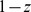. The fitness of mating between a male with bacterial strains of 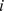 and a female with bacterial strains of 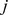 is generally described as 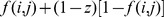.

A bacterial strain can be transferred by vertical transmission from a mother to offspring at egg stage with a probability of 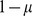, whereas it fails to be transferred with a probability of 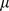. In the field, measurements of 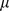 are typically low (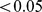). An individual host infected with the bacterial set 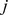 will produce eggs with 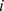 with a probability of 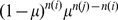 when 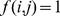. Note that our model assumes a diplodiploid sex determination system in the host insects, but the effects of CI can be quite different between diplodiploidy and haplodiploidy [31].

The occurrence frequency of CI depends on the mating pattern. Under random mating, CI occurs depending on the distribution of 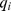. The average fitness of a female with the bacterial set 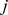 is 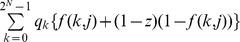. In contrast, under inbreeding, CI depends on 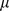. Mating partner tends to have the same infection status as themselves because the pair are possibly produced by the same mother. CI is less frequent under inbreeding than random mating because it occurs only under following conditions: (i) a mother lacks the bacterial strain(s) because of vertical transmission failure and (ii) a father carries the strain(s) because of their successful transmission from a common grandmother. It is difficult to formulate the exact effect of CI under inbreeding because the frequency in a previous generation is needed. For simplicity, we hereafter assume that 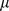 is sufficiently small to regard the average fitness of a female with the bacterial set 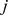 as 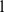. Let 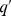 be the frequency of infected individuals in the next generation and 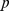 the probability of random mating. The difference equation of 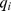 is described as follows:
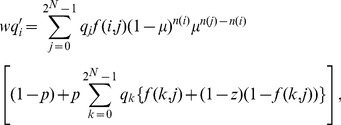
(1)where 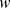 is a normalization factor that maintains the sum of the updated host frequencies as unity. Eq.(1) can be rewritten as follows:
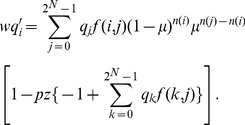
(2)


Eq.(2) is symmetrical for 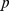 and 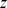, which means that the effect of 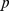 on the infection dynamics is equivalent to that of 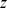. In addition, Eq.(2) reveals that the dynamics can be described using only two parameters, 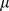 and 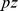. If two systems have the same value of 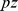, then the dynamics of these systems should be identical. If we use 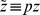 as the effective CI strength, then 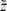 should correspond to 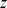 in the systems with the equivalent dynamics under complete random mating (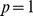). As a result, Eq.(1) can be rearranged to a more simple form using 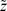 as follows:
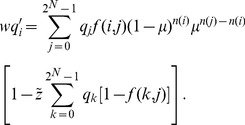
(3)


Eq.(3) is identical to the equation developed by Frank [18], but 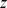 is substituted by 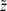. Frank suggested that if all the bacterial strains share the common parameters 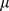 and 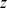, the polymorphic equilibrium solutions should be symmetric for the frequencies, i.e., the frequency of the hosts with the same number of strains should be identical. For example, if 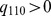, then 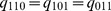 should be satisfied. Frank performed a reduction of Eq.(3) as follows:
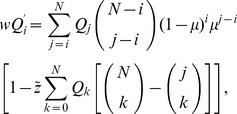
(4)where 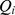 is the frequency of a type that carries 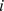 different strains, in which there are 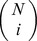 different combinations. We analyze Eq.(4) hereafter.

**Figure 1:**
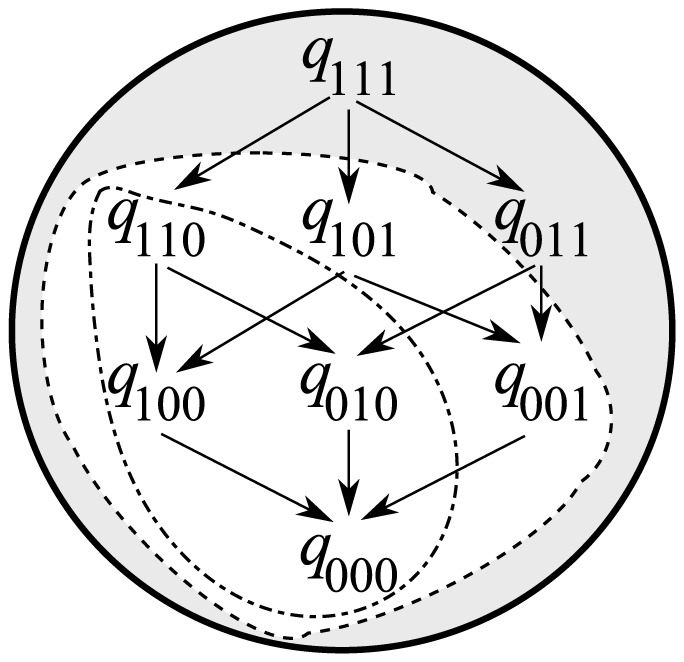
Schematic showing bacterial infection flows, where 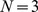. The host population comprises a set of triple infections, three sets of double infections, three sets of single infections and a noninfection set. The arrows represent vertical transmission failure. All sets can exist in the population (enclosed by the solid line) only when 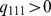. By contrast, when 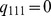, there are no stable states where the frequencies of more than one set of double infection has a positve value (enclosed by the dashed line). In that case, the host population eventually approaches a stable state that include two bacterial strains (encolosed by the dotted-dashed line). The proof was presented in [18].

Frank also proved that a polymorphic equilibrium, where both infected and uninfected individuals exist, can be stably maintained only if there are host individuals infected with all of the bacterial strains in the population [14]. In other words, if the largest 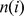 in the population is less than 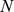, polymorphism should be lost and some bacterial strains will eventually fail to achieve infection until stable maintenance is possible (Figure 1). Frank stated that the presence or absence of individuals infected with all bacterial strains in a population determines the fate of the population. Therefore, we focus on the dynamics of the frequency of hosts infected with all of the bacterial strains in an 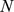-strain population, 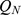.

Next, we analytically derive the equilibrium for the system [Eq.(4)]. We will first focus on simple cases with one or two bacterial strains in the population before generalizing the results to an arbitrary number of strains.

## Results

### Single infection

When 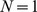, the system [Eq.(4)] can be represented as follows:
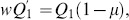
(5)where 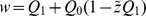 and the total frequency 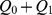 equals unity.

**Figure 2:**
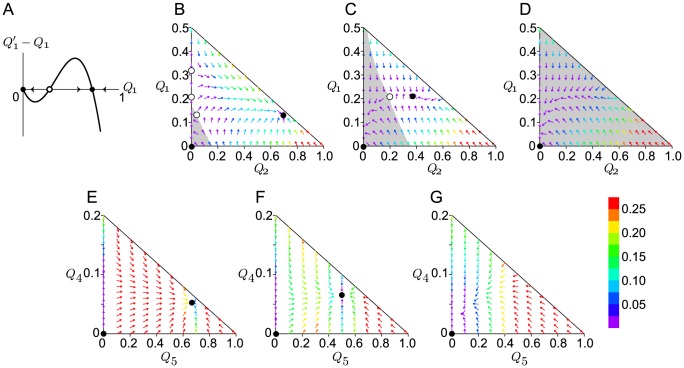
(A) Phase portrait where 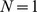, 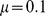 and 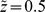. When the initial value of 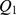 is less than the unstable equilibrium (open circle), 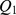 decreases toward zero (extinction of endosymbionts). Otherwise, 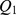 moves to a nonzero stable equilibrium point (closed circle on the right side). (B–D) Vector fields where 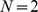 with 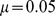 and 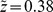, 0.24, and 0.20, respectively. The horizontal and vertical axes represents 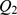 and 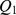, respectively. Each arrow represents the difference in 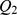 and 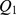 between a generation, 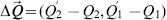. The color of an arrow indicates the magnitude of the vector. Solid and open circles indicate stable and unstable equilibrium points. The shaded areas depict the basin of attraction for the extinction of endosymbionts. (E–G) Vector fields where 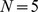 with 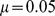 and 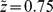, 0.50 and 0.25, respectively. Each arrow represents the difference of 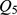 and 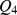 between a generation, 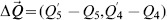.

Because 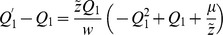, there are a maximum of three fixed points in the system: 
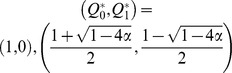
 and 
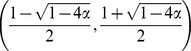
 where 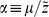. The phase portrait of the system is shown in Figure 2a. The second and third fixed points can exist only when 
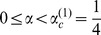
, because a saddle-node bifurcation occurs at 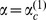. Thus, we can obtain the nontrivial fixed points except 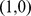 only for 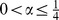. 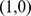 and 
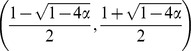
 are stable and 
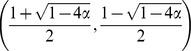
 is unstable (Figure 2a). The unstable fixed point shows the basin boundary. If the initial value of 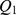 is below the unstable fixed point, then 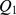 decreases asymptotically to 0, i.e. the population loses the bacterial infection. In contrast, if 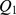 exceeds the boundary due to random drift or other accidental events, 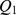 can converge towards 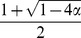. Note that because 
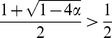
, whenever the bacterial infection reaches equilibrium, the frequency of infected individuals is certainly more than half.

### Double infection

In the case of 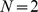, the system [Eq.(4)] can be represented as follows:
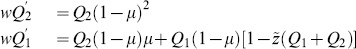
(6)where 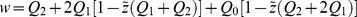 and the total frequency 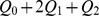 equals unity. This system has five fixed points as follows: 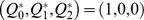, 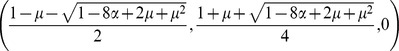, 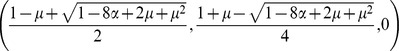, 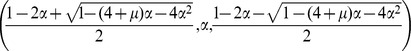 and 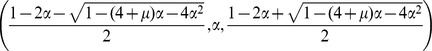. To examine the stability of the fixed points, we performed linear stability analysis for each fixed point. At 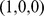, the eigenvalues of Jacobian are 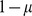 and 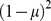. Because the absolute values of these eigenvalues are less than 1, the state where there are no infected insects are locally asymptotically stable. We numerically calculated eigenvalues for the other fixed points for any sets of 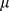 and 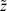, and concluded that only 
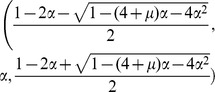
 was a stable fixed point and the others were unstable. This stable fixed point exists if 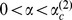, where 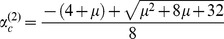. When 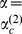, 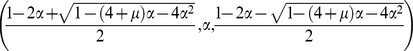 and 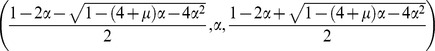 coalesce due to saddle-node bifurcation as well as when 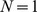. Because 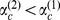 for any value of 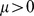, the range in which completely infected host can exist at 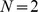 is narrower than that when 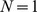.

**Figure 3:**
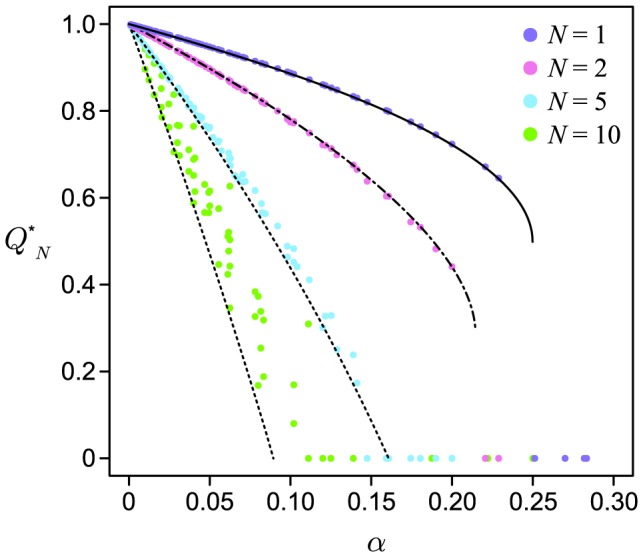
Comparison of 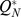 in the numerical (colored points) and analytical (black curves) solutions where 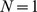, 2, 5 and 10. The parameters used in the simulations were selected from the ranges of 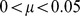 and 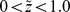. The convergence condition was 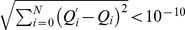. The analytically derived equilibria are shown by a solid line for 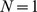 (
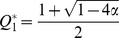
), a dash-dotted line for 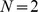 [Eq.(7)], and dotted lines for 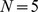 and 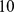 [Eq.(10)].

As stated in the previous section, we assume that 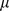 is a relatively small parameter. By substituting zero for 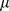 as a zero-order approximation, 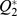 at the stable fixed point and the critical value of 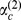 are given as follows:
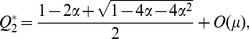
(7)

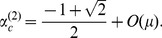
(8)The numerical simulation confirmed that this approximation is adequate and that 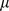 had less effcet on 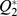 and 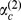 than 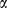 (Figure 3).

### 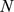-strain infection

Finally, we calculated the fixed points of Eq.(4) for arbitrary 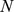. We assumed that 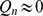, for 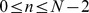 in an equilibrium state. Numerical simulations supported this assumption (Figure S1). For example, when 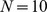, 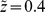 and 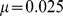, 95.5% of the population possessed 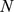 or 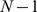 bacterial strains. The dominance of highly infected hosts was enhanced by the strain number 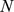. Thus, this assumption is more plausible when the system has a greater 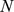 and a lower 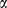. The reduced system is described as follows:
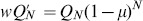
(9)where 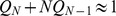, 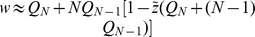. Because this is a one-dimensional system, the fixed points can be found easily, 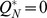, 
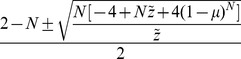
. If 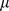 was regarded as a relatively small parameter, 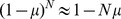. Then,
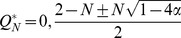
(10)which shows that the frequency of completely infected hosts depends only on 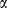 when 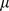 is sufficiently small. The difference in 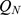 between one generation, 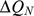, is represented as follows:
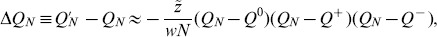
(11)where 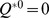, 
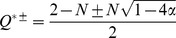
. If 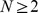 and 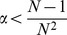, the order of the three fixed points is 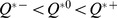 and 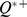 is a stable equilibrium.

Figure 3 compares 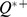 at various 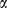 with the results calculated by numerical simulations for 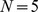 and 10. The approximated solution of 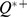 was generally consistent with the numerical results. In particular, the agreement was better when 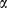 was sufficiently small.

Eq.(11) indicates that an exchange of stability, so-called transcritical bifurcation, should occur between 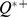 and 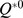 at 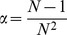. When 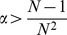, the order of fixed points is 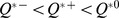 and the stable fixed point is 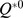. However, we did not observe a transcritical bifurcation for any parameter set of 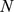, 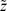 and 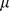. Instead, a discrete jump into 0 was observed before 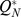 reached to 0, which implied that a saddle-node bifurcation also occured at 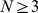 (Figure 3). The reduced system did not replicate the disappearance of the stable fixed point; however, the analytical solution of 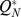 agreed with the numerical results until the discrete jump occured. In addition, The numerical simulation confirmed that when the bacterial strain number 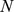 was sufficiently high, a discrete jump occured around 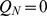. Therefore, with a large 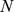 limit, the value of 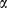 at which the transcritical bifurcation occurs is equal to 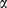 at the saddle-node bifurcation. Thus, we obtained the critical 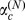 for a sufficiently large 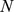 as follows:
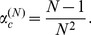
(12)


**Figure 4:**
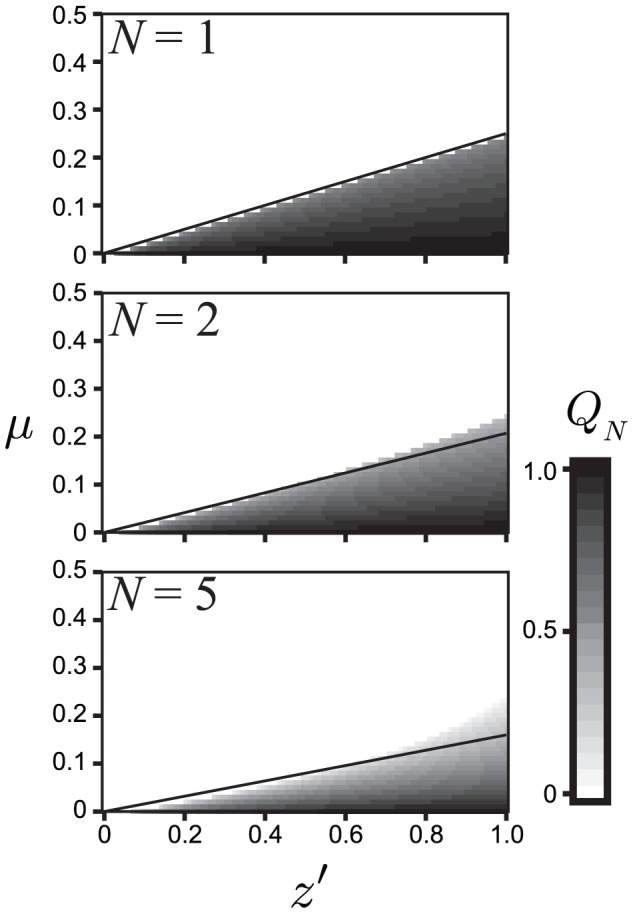
Distribution of the values of 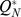 obtained by numerical simulations where 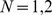 and 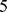. The dark highlights indicate the values of 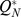. The lines in each figure represents the analytically derived critical value of 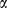.

**Figure 5:**
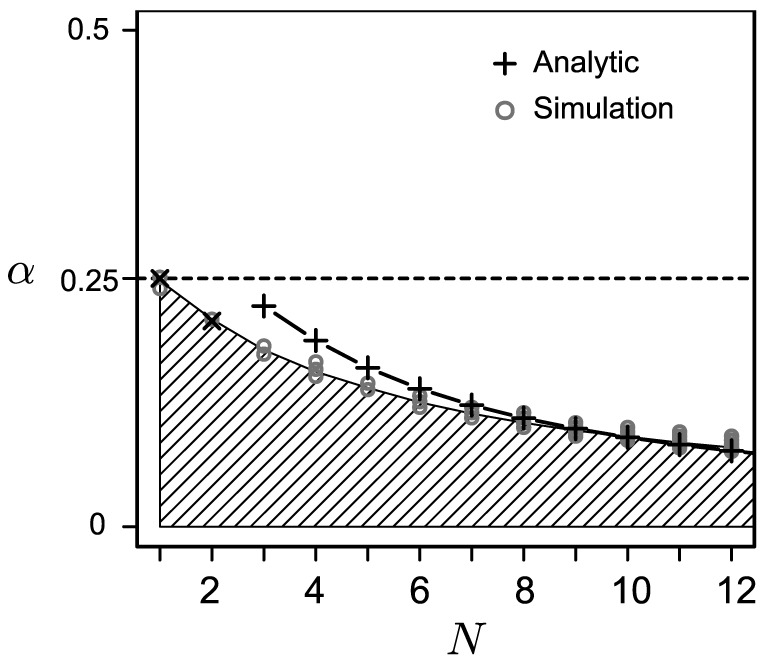
Threshold of 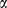 that allows bacterial infection. The numerical simulation confirmed that a host population where 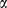 is in the shaded region could maintain infections of 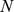 bacterial strains. The black crosses are the analytically derived thresholds. The horizontal dashed line is drawn at 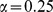.

We compared the analytically derived 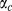 with numerical simulations. Figure 4 presents the numerically calculated 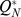 at various 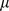 and 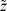 when 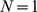, 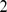 and 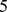. The lines in each figure indicate the analytically derived critical value of 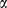, 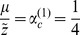, 
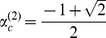
 and 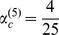. The lines successfully delineate the parameters where 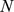 bacterial infections can be maintained. The consistency is reduced when 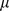 is greater because 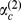 and 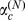 include 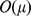 errors. In particular, the ranges of 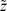 and 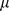 that allow infections with a complete set of bacteria are distinct. The value of 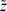 ranges from 0 to 1. In contrast, when 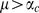, 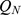 cannot have a positive value, irrespective of the value of 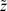. Figure 5 confirms the consistency between the numerical results and Eq.(12). The shaded area derived by simulation indicates the values of 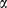 where 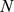 bacteria can infect a population. On the basis of the analytical results, we plotted the critical value of 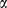 where 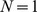 and 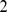, 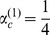, 
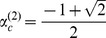
. For 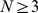, we plotted the critical 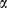 derived from Eq.(12). For larger values of 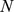, the analytical results approached the threshold derived by numerical simulation.

The inverse function of Eq.(12) defines the maximum number of bacterial strains that can infect a population, 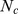, which is given as follows:
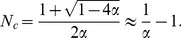
(13)

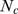
 is approximately proportional to 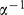. Therefore, it has a larger value with a lower value of 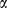.

## Discussion

In this study, we demonstrated the analytic equilibrium solutions of the frequencies of individuals infected with all the bacterial strains in a population with single, double and arbitrary 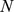 infections. Our results provide of a qualitative insight into the symbiotant bacterial dynamics, in constrast to recently developed models that quantitatively simulate specific experimental results [4], [32].

Our model used a parameter 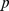, the level of inbreeding, from the perspective of host behavior. We revealed that 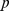 has a completely negative effect on CI. Endosymbionts manipulates host reproduction to maintain their own infections in host populations. In contrast, host insects can reduce the prevalence of endosymbiont infections by inbreeding. This result is consistent with previous numerical simulations of inbreeding [28]. Engelstadter et al. claimed that the infection rate decreases with increasingly inbred hosts because uninfected females increasingly mate with uninfected males, which leads to fewer incompatible matings. Our analytic calculation determined a linear relationship 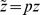, which clearly indicates the negative effect of inbreeding on the CI strength. However, this simple relationship does not appear to hold in some cases of stochastic models. Genetic drift in stochastic island model might have effects on inbreeding [29]. Our result is also consistent with the previous analytical result reported by Dannowski et al. [30]. This model focused on double infections by male-killing bacterial strains and analytically showed that higher level of host inbreeding leads to lower frequency of bacterial infections. Although their result was limited in the case of double infections, our result is applicable to arbitrary number of CI-inducing bacterial strains.

The analytically derived equilibrium depends mainly on one parameter, 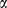. 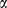 is a novel index that we defined in this study, which is the ratio of the probability of vertical transmission failure (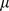) relative to the effective CI level (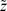). Previous studies have shown that these parameters are involved with the infection dynamics. Furthermore we demonstrated the simple relationship between these parameters, i.e., the bacterial infection dynamics are determined simply by a balance of both. The equilibrium frequencies of the fully infected host are at a higher rate when 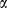 is lower (Figure 3). In this case, CI-inducing bacteria can select two alternative strategies to maintain and spread themselves: increasing the vertical transmission efficiency [16] and/or increasing the CI strength. There have been no reports of the differences among these strategies. Figure 4 presents the asymmetric relationship between 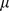 and 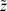. When 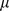 was higher than approximately 0.2, bacterial infections could not be sustained regardless of the value of 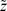. Thus, the vertical transmission rate has a threshold value for infection. In contrast, bacterial infections occur with any value of 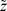 provided an appropriate value of 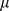 is selected on the basis on 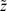. Thus, the vertical transmission rate determines the infection state more strongly than CI.

Eqs(12) and (13) represent the relationship between the maximum number of bacterial strains and the bacterial parameters. Considering a host population containing 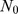 bacterial strains, if 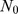 increases to 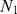 through the horizontal transfer of a bacterial strain, the critical valule of 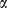 decreases from 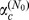 to 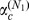. Provided 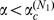 is satisfied, the 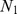-infecting population will be maintained. Otherwise, the infection cannot be maintained. For example, a population where 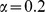 can maintain three strains at most because 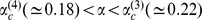. If the population actually contains the maximum number of strains, the host population is “saturated” with bacteria, i.e. other strains will fail to invade the host population. The parameters are expected to have the same value in our model. Therefore, the frequency of the rarest strain necessarily declines to zero. A higher 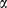 value than that of other strains may be needed for a new strain to invade the saturated hosts.

Our theoretical results suggest that the equilibrium frequency of completely infected hosts decreases as 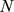 increases (Figure 3). In addition, the range of 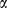 where bacteria can be maintained becomes narrower as 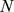 increases (Figure 5). The severe conditions for multiple infections are caused by the increased possibility of vertical transmission failure the offspring by any strains. 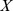-strains that infect a individual can successfully transmit all the strains to the offspring with a lower probability than 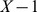 strains. Consequently, these results support a noble idea of *Wolbachia*-induced speciation. Suppose that 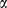 is enough large to maintain single infection but not double infection (0.207–0.25), and endosymbiotic strains, A and B strains, infect some insects in two different geographic regions respectively. The infections in both regions will spread until a hybrid zone is produced. Then, horizontal transfer makes double-infected individuals of A and B strains. However, the double infection must be lost because 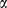 is too low to maintain the both infections. Therefore, the hybrid zone will be unstable and post-zygotic speciation will be occurred between A-infected and B-infected individuals.

**Figure 6:**
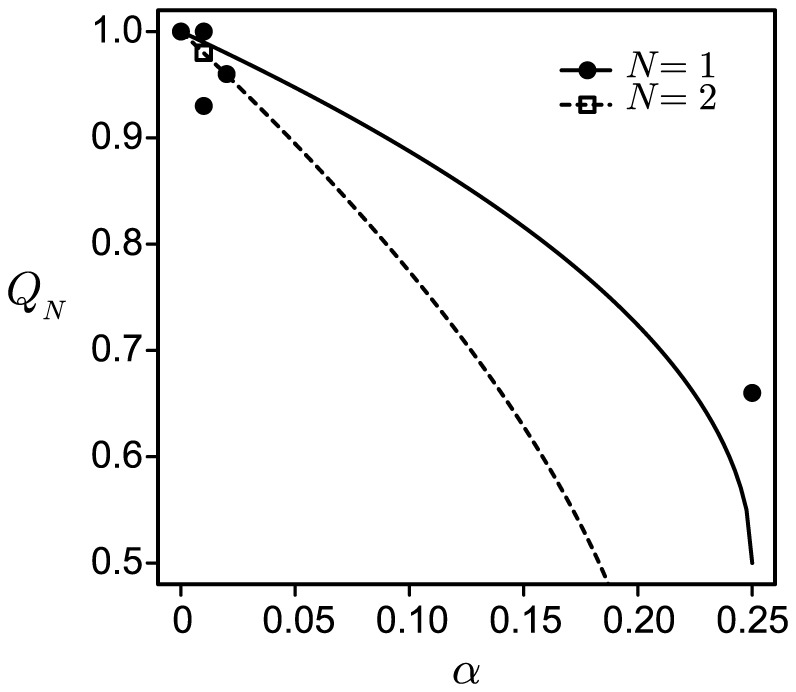
Comparison between the analytic equilibria for 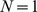 and 2, and the reported data. The solid and dashed lines represent the analytic estimates for 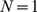 and 2, respectively. The closed circles and an open square indicate each single infection and double infection dataset, respectively. The values of 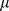 and 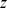, and the references are shown in table S1.

To confirm that our results were consistent with experimental data, we compared the predicted equilibria with the values of CI-inducing *Wolbachia* and *Cardinium* obtained using natural populations or an artificial line (Figure 6, Table S1). No data were available for triple or higher multiple infection. Therefore, only five single infections and one double infection were used for reference. No data were available on the inbreeding frequency of the insects studied; hence, 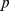 values of all were assumed to be zero, i.e. completely random mating. The experimental values were close to the predicted lines. Thus, our results can be used to estimate the frequency of infected hosts at equilibrium using the parameters (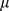 and 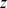) as well as for estimating the parameters that are often difficult to be measured.

In this study, we aimed to provide a qualitative outline of the dynamics of multiple infections by introducing an analytically solvable model. By ignoring quantitative accuracy, our model sheds light on the mathematical structure related to the multiple infection dynamics. However, we introduced several assumptions to develop a solvable model, which should be eliminated to allow more quantitative comparisons using experimental data. First, we assumed that a common transmission rate or CI level were shared among bacterial strains. If the bacterial parameters are heterogeneous, a bacterial strain with a lower 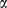 is more likely to be maintained than other strains. Indeed, some experiments indicate that the CI range is too broad to be regarded as the same value among different strains. For example, the CI levels of two *Wolbachia* strains (wBruCon and wBruOri) that infect *Callosobruchus chinensis* are variable [33]. The egg-hatching rate between double-infected males and wBruCon-infected females declined to 0 whreas that between double-infected males and wBruOri-infected females decreased to 0.62 due to CI. Another example is *Drosophila simulans* infected with four *Wolbachia* strains in Madagascar. The CI levels of the hosts vary between 0 and 1, depending on specific crosses [34]. Second, the effect of CI was assumed to be bidirectional for any combination of strains. However, it is known that some strains can compensate for the effect of CI by substituting for an uninfected strain [35]. To address more complex CI patterns, case-by-case models should be developed. Third, we ignored the bacterial density in a host by categorizing the hosts as infected or uninfected. However, it has been reported that the number of bacteria in a host insect depends on the specific combination of bacterial strains in multiple infections [36]. Thus, the bacterial density might affect the transmission rate and CI strength. The number of bacteria in the hosts should be used as a variable in individual-based models to examine the effects of bacterial density. Forth, we assumed that the geometrical structure of infected hosts was uniform. Thus, we did not consider the desity of the hosts. The crowding of hosts might occur by chance locally, which may cause increasing numbers of bacterial strains to be stably maintained. This effect should also be examined in an individual-based model. Finally, we roughly approximated the frequencies of hosts infected with 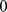 to 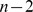 strains as being equals to 0 to calculate the equilibria for 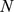 arbitrary strains. A more rigorous approximation method would reduce the error between the numerical and analytical results. The elimination of these assumptions needs to be addressed in future studies.

## Supporting Information

Figure S1
**Dominancy of highly-infected individuals.** (A) An example of distribution of frequencies 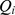 in equilibrium state. The frequencies were obtained by simulation with 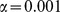 (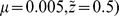 and 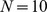. (B) The ratio of 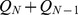 in equilibrium state where 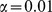 (closed circles) and 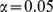 (open circles).(EPS)

Table S1 **The values of parameters and the frequency of completely infected hosts in the field.** These values were reported in the references listed above.
(EPS)
